# Built Environment Interventions to Increase Active Travel: a Critical Review and Discussion

**DOI:** 10.1007/s40572-019-00254-4

**Published:** 2019-11-26

**Authors:** Rachel Aldred

**Affiliations:** University of Westminster, Westminster, UK

**Keywords:** Cycling, Infrastructure, Built environment, Active travel, Physical activity, Walking

## Abstract

**Purpose of Review:**

To review the literature on built environment interventions to increase active travel, focusing on work since 2000 and on methodological choices and challenges affecting studies.

**Recent Findings:**

Increasingly, there is evidence that built environment interventions can lead to more walking or cycling. Evidence is stronger for cycling than for walking interventions, and there is a relative lack of evidence around differential impacts of interventions. Some of the evidence remains methodologically weak, with much work in the ‘grey’ literature.

**Summary:**

While evidence in the area continues to grow, data gaps remain. Greater use of quasi-experimental techniques, improvements in routine monitoring of smaller schemes, and the use of new big data sources are promising. More qualitative research could help develop a more sophisticated understanding of behaviour change.

## Introduction

This review examines recent literature on built environment interventions and their impact on active travel. Its aim is to assess the current state of the research field, including methodological and data availability issues affecting the quality of the evidence. Structurally, it begins with a summary of recent evidence, before moving on to consider key challenges, focusing on the increasingly popular ‘natural experiment’ approach. It ends by suggesting pathways for future research to improve the quality of future evidence.

‘Built environment interventions’ here means changes such as the provision of cycle infrastructure, the pedestrianisation of shopping streets, or the creation of local parks which create or improve active travel routes. Literature generally refers to active travel as walking and/or cycling, rather than (for instance) jogging or use of newer semi-active or electric-assisted modes (such as e-scooters). Public transport is usually seen as non-active, although trips to access public transport hubs may involve walking or cycling.

## Current Knowledge on the Topic

Much cross-sectional literature examines built environment factors correlated with levels of walking and cycling. Broadly there are two types of factors: (i) qualities of the built environment thought to improve walking and cycling experiences (wider footways, cycle paths, less air pollution, etc.), and (ii) the presence of relevant destinations within a reasonable distance (particularly for walking, whose range is shorter). While this evidence suggests that good active travel environments and destination proximity are associated with more walking and cycling, association does not necessarily imply causality [1–3].

The same problem exists for the large number of studies exploring preferences for different types of environment, in relation to walking or cycling. This broadly supports the cross-sectional literature: people can distinguish between and express preferences for built environment factors seen as better for walking and cycling, such as wide footways and protected cycle infrastructure [4, 5]. However, again we are faced with a causality problem. People may say they would like public parks, wide footways, reductions in motor traffic, cycle tracks, and so on, and even that such changes would encourage them to walk or cycle.

But will this happen? Even if causality exists, pathways to change may not be transferable. For instance, it seems plausible that many people cycle in the Netherlands primarily because of a supportive environment, including but not limited to high quality cycling infrastructure [6]. However, for policy-makers in low-cycling countries such as the UK, the key question is whether, if we build cycling infrastructure now, in a UK environment, we will see more cycling; or whether factors such as cultural barriers to cycling mean that change is out of reach. Given the intense controversy that may be associated with built environment interventions, particularly pro-cycling changes in lower-cycling contexts [7, 8], there is a need for evidence focusing on interventions and change.

Recently we have seen a rise in intervention studies (such as longitudinal studies) and systematic reviews assessing these [9, 10], helping to fill the gap. In general, studies find the expected associations, although some have not done so. More and better studies are still needed, particularly for walking infrastructure [11]. Due partly to sample size, there is often relatively little consideration of subgroup effects, although studies suggest there may be differences at least in the strength of preferences expressed for ‘good’ improvements. For instance, cycling research finds that infrastructure separated from motor traffic is perceived as particularly important by women and those cycling with children [5, 12]. Walking research has similarly highlighted potential differences in perception in low-income areas, including related to micro-scale factors that may be related to (perceived) risk of crime [13].

To summarise, current evidence increasingly suggests that changing the built environment can affect levels of active travel. Increasingly studies cover actual interventions, rather than only cross-sectional associations, preferences or participant views about what would influence their future behaviour. However, there is a need for more studies with gaps remaining particularly for walking, and for evidence around distributional impacts and subgroup differences. The next section of the paper discusses methodological issues affecting the field, including strengths and tensions arising from its interdisciplinary nature.

## Discussion

The literature on built environment interventions and active travel uptake exists at the intersection of health and transport planning. The different disciplinary traditions have implications for what we value and measure, and differences in approach to evidence and key outcomes are discussed further below. However, transport and health agendas have come together in recent years, at an academic and a policy level. While public health is interested in any physical activity, it is increasingly recognised that our best hope of reducing dangerously high levels of physical inactivity is to build active travel into everyday life, rather than it being a separate activity that people must take time out of their lives to complete. Conversely, transport authorities are increasingly seeing health as a core part of their remit, such as the ‘Healthy Streets’ approach developed by Transport for London [14].

### Appropriate Evidence Standards

Transport and health fields have different traditions within which to view monitoring and evaluation. Within transport, monitoring and evaluation have traditionally depended on estimating or measuring impacts on car users, usually time savings, to the exclusion of many other issues from environmental damage to delays to pedestrians [15]. This focus has in recent years been challenged. Many cities are seeking to improve walking and cycling infrastructure, and hence to increase levels of walking and cycling. However, they are not helped by traditional planning tools, which offer much sophistication in assessing motor traffic throughput along links and junctions, but little sophistication in estimating how schemes might affect walking and cycling uptake.

Transport authorities do often conduct their own monitoring of active travel schemes, and they or their consultants write reports on outcomes. When evaluated by academic standards such research (understandably) suffers by comparison to ‘gold standard’ evidence. Known issues include the lack of longer-term follow-up, and a lack of control sites or comparators. This is crucial because weather affects walking and cycling levels, so without a control strategy, a year-on-year change in active travel might simply be due to changes in weather. In the ‘grey literature’ there is frequently a failure to effectively measure changes in active travel. Instead, organisations may use count data as a measure of new trip generation, whereas it could simply represent diversion [16•]. The academic transport literature has traditionally often taken a ‘case study’ approach where ‘good (occasionally bad) practice’ examples are described and analysed in depth. While often providing useful in-depth insight into policy packages and discourses [17], this approach does not lend itself to evaluating and generalising about the impacts of specific interventions.

Rising interest in this area among public health researchers has encouraged the identification of a range of potential biases associated with existing grey or academic literature assessing impacts of interventions [18]. However, adopting medical standards of evidence is not always straightforward for built environment interventions, with randomised controlled trials generally not feasible. The researcher is usually unable to control allocation of individuals into groups, whereas this may be possible^1^ for individual- or organisational-level interventions (for instance, provision of cycle training, or workplace measures). Political obstacles and controversies frequently affect the introduction of built environment changes, with lengthy consultation processes meaning many residents are aware of new interventions before they happen, often through controversy and local press coverage [19].

While not necessarily a bad thing (knowing that a new facility exists is likely to be part of its pathway to impact) this does make traditional quality measures such as blinding participants to their treatment group inappropriate. Avoiding the ‘placebo effect’ has traditionally been fundamental to design of medical and public health interventions: but what might a ‘placebo effect’ even mean in the context of built environment? We would need a clearer understanding of the relative contributions of pathways to effects to theorise this. Some fields have used the ‘placebo’ concept to understand pathways to impact in their area. For instance, within environmental psychology a ‘placebo effect’ has been identified whereby an ‘eco-label’ rating affects perceptions of product performance [20]. If we can better understand ‘what matters’ for walking and cycling uptake, we would be better placed to develop study designs that can separate this from any ‘placebo’ type impacts; if indeed the ‘placebo’ concept proves useful for the field. We might instead want to talk about a distinction between direct benefits from changes to infrastructure or facilities, and behaviour change induced through broader cultural processes whereby people are influenced by (hearing about) such changes, because they send a signal that active travel is important.

### Objectives, Metrics, and Methods

Another issue affecting the interdisciplinary evidence base relates to the outcomes prioritised and valued in different fields. While both public health and transport planning are interested in active travel, the supplementary objectives may vary. For transport planning, end goals are generally transport-focused. While some interventions may primarily improve conditions for existing cyclists or walkers [21], this may not constitute a failure for the transport authority, as improving journey ambiance for travellers is an important part of their remit. However, often a key end goal is reduction of car-driver trips; and sometimes, cities fear abstracting from public transport [22], given that they fund and/or run extensive public transport systems. By contrast, from a public health perspective switching medium length trips from bus + short walk to cycling might create substantial benefits, due to the increased physical activity.

These different foci lead to different methods for measuring behaviour change. Public health literature is more sceptical of self-reported physical activity [23], due for instance to recall concerns. By contrast, transport planning has long relied on travel diaries to measure use of different modes, and thus studies within this tradition more often use subjective measures. Traditionally these travel surveys have focused on walking or cycling for a purpose or to a destination, rather than walking or cycling purely for pleasure or leisure (e.g. walking the dog in a park). The extent to which the use of subjective or objective measurement matters will vary. Travel diaries are likely to be more reliable for recording cycling and main mode walk trips than for short walks made as part of multi-stage trips; which some travel surveys do not even seek to capture. Subjective recall of physical activity is likely to be worse than of travel.

Use of objective measurement, either through ordinary operation of a smartphone or specific apps, looks increasingly promising for measuring use of different modes [24–26]. This has been found to be less accurate for measuring amount of physical activity [27] compared with bespoke devices [28], which while more traditional are more expensive and can mean higher participant burden. Measurement accuracy is however improving [29, 30]. As this continues, the existence of ‘big data’ opens a door to conducting large scale studies by piggybacking onto data collection for other purposes (e.g. health and fitness apps), if ethical and access issues can be resolved. A recent article [31] suggests that aggregated data such as that from the Strava app (packaged for city use as Strava Metro, as a proprietary, paid-for product) can help evaluate the impact of specific infrastructure changes, while they are less useful for making broader inferences on change across a region, due to differential take-up (middle-aged adult men being disproportionately represented). Such data has been recently used to estimate the impact of new cycle infrastructure in Glasgow [32••].

### Natural Experiments in the Built Environment Field and Their Challenges

While RCTs may not be appropriate for built environment interventions in general, epidemiological methods are increasingly contributing to their evaluation. Natural experiments in particular are increasingly used to study various types of intervention, including those related to cycling and walking environments [33••]. By treating an intervention as an experiment to be evaluated using a control (unexposed) and intervention (exposed) group, the major strength of the method is that it offers a quasi-experimental approach that can allow us to distinguish between impacts of a specific intervention and changes due to other factors. A major weakness is that because individuals are not randomly allocated to control and intervention groups, differences may exist beyond the presence of the intervention. This is very possible given potentially controversial interventions where—for instance—political support may shape where changes do or do not happen.

A further challenge for these and related types of intervention study is that often interventions are multi-faceted; indeed, policy-makers are recommended to introduce multi-faceted interventions [34] as more likely to succeed. A possible response to this is to define intervention areas widely, which has the benefit of making the use of existing data easier (whether through administrative data or new big datasets). Using secondary data can substantially reduce study costs—important given the often-high cost of collecting new data to evaluate interventions. It can enable the analysis of more data than could be typically collected through a new longitudinal study, hence facilitating the analysis of interaction effects—a gap in the evidence as discussed above. Disadvantages are the inability to discriminate between specific interventions and that existing data may not well measure travel behaviour. In a UK-based study, town-wide cycling initiatives were evaluated using administrative data from the decennial Census, completion of which is mandatory [35]. This provided (changes in) travel-to-work data for almost the entire population. However, the data only relates to commuting (less than one in five of all trips) and uses a ‘habitual behaviour’ question; although the resulting measures of cycling do correlate well with those derived from travel surveys.

A second, related, challenge covers how we characterise interventions in the first place. City authorities often brand interventions or use ill-defined terms to describe them; for instance, as ‘cycle superhighways’, ‘complete streets’, ‘bicycle boulevards’, or ‘traffic calming’, and these terms are often then also used in academic literature. All are somewhat amorphous and may represent very different route environments or interventions even within the same city, let alone in different cities, countries, and regions. In the London, UK, case, a cycle superhighway variously might imply a wide one- or two-way cycle track separated from motor traffic and pedestrians; a designated route along supposedly quiet side streets; a blue painted cycle lane; or a shared bus lane.

The stated preference literature suggests these different facility types have very different levels of attractiveness to users [36], which may translate into differences in uptake. Analysing the impacts of London ‘cycle superhighways’ as a type of infrastructure may thus tell us relatively little about characteristics of the route environment that can increase levels of cycling. More broadly there is often a problem with generalisability. Where studies only look at commuting, can we assume that there will be changes in other types of travel? Can we assume that results of an intervention in one region, country, or city, are likely to happen in another, with a different surrounding context?

There is also the question of distinguishing ‘carrot’ and ‘stick’ interventions. These colloquial names for interventions aimed at (i) increasing active travel and (ii) discouraging driving are poorly chosen. Interventions that offer better conditions for walking or cycling often necessarily discourage driving: for instance, by re-purposing car parking spaces as pocket parks or cycle infrastructure. Limited amounts of space and time, and competing modes, mean that this is often unavoidable. Yet to what extent, for instance, do wider footways increase walking uptake (if they do) by comparison with a reduction in motor traffic entailed by the associated reduction in space for cars? At present, there is limited evidence specifically how reducing space, time, or facilities for motor traffic affects walking and cycling. Within transport studies, some work deals with its impact on driving. A 1998 review concluded that if space for cars is cut, much motor traffic will often simply disappear [37]. ‘Traffic evaporation’ may be made up of a range of behaviour modifications; from changing journey time, destination, or route; to simply not making a trip or ordering online; to combining trips differently; to shifting a trip to walking, cycling, or public transport. Here we are particularly interested in mode shift, for which much of the evidence tends to use lower quality study designs such as uncontrolled case studies [38].

Even where interventions are easier to define, there is a broader challenge of identifying the population exposed to an intervention, given travel is often *to* somewhere (and yet, as above, that ‘somewhere’ can potentially change for many types of trip). Studies often use distance to specific interventions, such as new cycle routes or walking/cycling infrastructure [39, 40], although the impact of distance may vary depending on location of key destinations. For instance, a route from suburbs to centre might have the highest impact in the middle, if most trips are headed towards the central area. It may be less useful for those who live in a central area and do not need/wish to journey to the suburbs, or for those living in the far suburbs for whom the trip to the centre is too far to cycle. A single distance measure implicitly assumes these differences do not matter.

A study of London ‘mini-Holland programmes’ [19] in three boroughs (municipalities) used a subjective approach to exposure, asking local stakeholders in each borough to define ‘high-dose areas’ (repeated annually) within their borough, where they thought interventions might have a direct impact on travel behaviour. This allowed analysis to draw on expert knowledge of how, for instance, a new route might serve some areas through which it passed better than others (for instance, because of the differing quality of existing infrastructure to which the route connected). The study found an increase of 41 minutes weekly active travel (mostly composed of walking) after 1 year among those living in the high-dose area. However, there was no statistically significant increase among those living within low-dose areas in intervention boroughs where no local changes had been made. Hence there seemed to be a clear impact associated with specific built environment changes, rather than simply from the broader borough-level programme in general (e.g. through borough-wide publicity or promotion). Had the study used only borough-level analysis, this change would have been missed.

Despite all the methodological challenges, the natural experiment approach provides one model for how public health approaches can be adapted for use in studying the built environment. These studies have advanced the evidence base by providing better evidence around causality than that which can be delivered through cross-sectional studies, case study research, and stated preference studies. They are not the only useful studies, and I will suggest in the conclusion that they need to be supplemented, including by innovative qualitative work, and by attempts to develop more rigorous classifications of intervention typologies and their likely effects.

## Conclusion: What’s Next?

It is usual for such reviews to conclude with a call for more, high-quality studies. This would indeed be useful. We still need more natural experiments and other high-quality studies, particularly covering subgroup differences and impacts on walking. However, other work needs to be done to strengthen the underlying models shaping our understanding of travel behaviour [41]. Much work relies on an assumption that intention precedes action; however, a recent study of behaviour change during the London Olympics found that over half of those who were not considering making a change did go on to do so [42]. Investigation of mode shift often considers it as a one-time process (did you change from the car? From the bus? and so on), yet there may be knock-on impacts of such changes. For instance, if metro users shift to walking (after a new path is introduced), this might reduce crowding, and thus encourage drivers to then switch to public transport. More broadly, people who start walking or cycling more might then change where they shop or socialise, finding that local shopping is more convenient and ‘big box’ retail less so. We know relatively little about these processes, meaning that there is a need for more in-depth, qualitative research about responses to interventions.

This understanding could in turn help shape how and what we measure, and length of follow-up, often relatively short even in academic studies. The London study of ‘mini-Holland schemes’ referred to above [19] found no change in car use after 1 year, suggesting that increased active travel represented additional journeys, rather than having replaced car travel. However, after 2 years within specific high-dose areas changes were found [43], suggesting that people initially may have increased active travel trips without cutting car use (e.g. more local leisure walking) but over time this settles down and new active travel trips replace some car trips. However, without qualitative investigation into these changes, it is difficult to understand these trajectories and how they might vary.

Research could usefully do more to tease out and separate the possible impacts of different components of the built environment that affect walking and cycling. Given the variety of factors affecting how, when, and where we travel, this seems like a big ask, especially given cross-cultural variation in design of different aspects of the street environment. However, there is a role here for relatively low-cost preference-based research. Asking people what types of environment they would prefer to walk or cycle in does not necessarily tell us about take-up. It might, however, allow us to better understand what components of a walking or cycling environment are perceived as more or less important, and why, by different sub-groups and in different contexts. This in turn can allow a more systematic approach to categorising and evaluating interventions. The ability to use visual material (photos, computer-generated images, videos) within online or computer-assisted surveys means that such research can be done in a much more sophisticated manner than in the past, where people were simply asked their views on ‘a cycle lane’ [44].

Finally, more work could be done considering the extent to which medical research models could and should be adapted to deal with transport research and policy-making. The involvement and contribution of public health disciplines has substantially enhanced the field, highlighting the sometimes biased and/or limited nature of evidence within the transport field. But should transport research be adopting an identical hierarchy of evidence to that developed within medical science? For individual-level interventions, presumed to operate independent of knowledge and belief (paradigmatically, a pill), the double-blinded RCT is seen to represent the pinnacle of research excellence, by isolating the impact of the intervention from ‘irrelevant’ context.^2^ Yet does the transport field, where beliefs and perceptions are not simply bias but part of the ways in which interventions (do not) work, need different hierarchies; different ways of conceptualising relationships between different factors, and pathways to change?

## Footnotes

1 Although not usually done, as transport authorities may not appreciate the value-added of randomising and staggering introduction of such measures.

2 Although medical RCTs have often only recruited a sub-set of the population, seeing some people’s characteristics as more inherently biasing than others’.
